# Stereotyped behaviors: behavioral markers of brain dysfunction and welfare impairment in animals

**DOI:** 10.3389/fvets.2026.1797422

**Published:** 2026-03-17

**Authors:** Jinyue Zhang, Weiguo Cui, Shuai Zhao, Guoan Yin

**Affiliations:** 1 College of Animal Science and Veterinary Medicine, Heilongjiang Bayi Agricultural University, Daqing, China; 2 Key Laboratory of Exploration and Innovative Utilization of White Goose Germplasm Resources in the Cold Region of Heilongjiang Province, Daqing, Heilongjiang, China

**Keywords:** Animal Welfare, behavioral addiction, Brain function impairment, neuroplasticity, stereotyped behavior

## 1 Introduction

Stereotyped behaviors are repetitive and unchanging behaviors that occur frequently in specific contexts, appear to lack immediate purpose or function, and are commonly observed in animals housed in captivity or under intensive rearing conditions. In animal welfare science, stereotyped behaviors are widely recognized as external manifestations of compromised welfare in adverse environments (1, 2). Among domestic animals, pregnant sows are a valuable model for studying stereotypic behavior because of their apparent sensitivity to feeding patterns, spatial constraints, and environmental complexity. Under conditions of confinement or barren housing, sows frequently develop oral and locomotor stereotypies, including sham chewing, bar biting, and repetitive rooting (3). Notably, stereotyped behaviors often persist even after improvements in housing conditions, suggesting that once established, these behaviors may become partially decoupled from the original eliciting environment. Empirical studies indicate that environmental enrichment may reduce the frequency of stereotypy but often fails to completely abolish well-established repetitive behaviors, implying longer-term neural or behavioral stabilization mechanisms (4–7). In parallel, human neuroimaging studies have demonstrated strong associations between alterations in cortico–basal ganglia white matter connectivity and restricted repetitive behaviors, particularly in neurodevelopmental disorders (8). Collectively, these observations indicate that stereotyped behaviors reflect more than superficial behavioral abnormalities. In this Opinion article, we contend that stereotypy represents a cross-species neurobehavioral phenotype arising from convergent dysfunction within cortico-striatal-thalamo-cortical (CSTC) circuits. From this perspective, stereotyped behavior may be interpreted as a behavioral manifestation of impaired neural plasticity and altered brain function, with direct implications for the assessment of animal welfare. Stereotyped behaviors are repetitive and unchanging behaviors that occur frequently in specific contexts, appear to lack immediate purpose or function, and are commonly observed in animals housed in captivity or under intensive rearing conditions. In animal welfare science, stereotyped behaviors are widely recognized as external manifestations of compromised welfare in adverse environments.

## 2 Cross-species stereotypy and convergent neural circuitry

Stereotyped behaviors have been documented across a broad range of species, including rodents, farm animals, and humans, suggesting that this phenomenon reflects conserved vulnerabilities in neural systems governing behavioral control (9). Experimental models of stereotypy encompass environmentally induced, pharmacological, genetic, and pathology-related paradigms (10). Despite heterogeneity in induction methods, converging evidence from pharmacological, genetic, and neurodevelopmental models implicates dysfunction within CSTC circuitry. The CSTC loop plays a central role in behavioral selection, inhibitory control, and the balance between goal-directed and habitual action systems. Disruption of pre-frontal regulation over striatal output biases behavior toward inflexible, habit-dominated patterns (11–14). When top-down control from the pre-frontal cortex over the basal ganglia is compromised, behavior becomes increasingly automatic, repetitive, and insensitive to environmental feedback (11). Cross-species evidence indicates that the severity of stereotyped behaviors is often associated with structural and functional alterations in cortical–striatal connectivity, a relationship that has been observed both in animal models and in humans exhibiting restricted repetitive behaviors (8). Table 1 summarizes representative animal models of stereotyped behavior across species. Although the overt expression of stereotypy varies—from oral movements in pigs to repetitive locomotion in rodents—these models converge on shared neurobiological characteristics, including dopaminergic dysregulation, impaired cortical inhibitory control, and maladaptive engagement of habit-related striatal pathways. This convergence supports the interpretation that stereotyped behavior constitutes a unified neurobehavioral syndrome rather than a collection of unrelated abnormal actions.

**Table 1:** Selected animal models of stereotyped behavior.

**Model type**	**Induction conditions**	**Typical stereotyped behaviors**	**Representative neural alterations**	**Species**	**Ref**.
Environmental deprivation	Barren or impoverished housing	Repetitive jumping, flipping	Reduced cortical regulation, altered basal ganglia metabolic activity	Deer mouse	(28)
Cage-induced stereotypy	Standard laboratory housing	Route tracing, bar mouthing	Striatal dopamine dysregulation, enhanced habit circuit engagement	Mice	(29)
Chronic confinement	Gestation stalls; feed restriction	Sham chewing, bar biting	Chronic stress activation, CSTC imbalance, elevated cortisol	Sows	(3, 20)
Dopaminergic genetic model	DAT knockdown	Sequential super-stereotypy	Hyperdopaminergic striatal signaling, impaired inhibitory control	Mice	(30)
Psychostimulant-induced model	Amphetamine or cocaine exposure	Repetitive sniffing, oral movements	Corticostriatal loop overactivation, sensitized dopamine transmission	Rats	(10, 31)
Autism-related model	Genetic background (BTBR)	Repetitive grooming	Frontal-striatal connectivity alterations, reduced behavioral flexibility	BTBR mice	(32)

## 3 Pathological neuroplasticity underlying stereotyped behavior

Neural plasticity enables organisms to adapt behavioral strategies in response to environmental demands; however, prolonged stress, deprivation, or repetitive stimulation can drive maladaptive forms of plasticity. Under chronic adverse conditions, synaptic and circuit-level remodeling may progressively stabilize rigid behavioral patterns, thereby favoring the emergence of persistent stereotyped actions (15). At the circuit level, stereotyped behaviors are consistently associated with excessive engagement of sensorimotor striatal loops accompanied by diminished regulatory input from the pre-frontal cortex. This imbalance biases behavioral control toward habit-dominated processes and substantially reduces behavioral flexibility (6). Alterations in glutamatergic transmission and dendritic spine remodeling have been reported in several models of repetitive and compulsive behavior. Dysregulated corticostriatal glutamatergic signaling is thought to impair behavioral updating and flexibility, while structural spine changes in the striatum may stabilize maladaptive habit circuits (16–19). Farm animals subjected to long-term confinement may provide a useful long-term naturalistic context for examining mechanisms parallel to those observed in laboratory models, especially as certain behavioral features such as persistence and resistance to change have been documented across diverse species. While direct neurobiological investigations in farm animals remain limited, physiological and behavioral data from long-term confined sows suggest chronic stress-associated systemic alterations that may plausibly extend to neural circuit remodeling (3, 20). Furthermore, high levels of maternal stereotypy have been linked to long-term emotional and behavioral alterations in offspring, suggesting that the neural consequences of stereotyped behavior may extend across generations, potentially through epigenetic mechanisms (21). Taken together, these findings support the interpretation that stereotyped behavior may reflect a relatively stable neuroplastic adaptation that persists beyond the initial stressor, although accumulating evidence from epigenetic and circuit-level studies suggests that such changes may retain a degree of plasticity and potential reversibility under targeted interventions (15, 22).

## 4 Stereotypy, habit bias, and brain dysfunction

From a behavioral standpoint, stereotyped actions are characterized by high degrees of automaticity, reduced sensitivity to feedback, and marked resistance to change—features that closely resemble habitual and compulsive behaviors. Within the continuum extending from goal-directed actions to compulsive or addictive behaviors, stereotypy appears to occupy an adjacent position (12, 13). Although stereotyped behavior does not depend on exogenous substances, it may emerge from persistent abnormal activation of neural circuits mediating reward processing and habit formation. Dopaminergic signaling within the striatum plays a critical role in this process by facilitating the transition from flexible action selection to rigid habit expression (23). In pigs, pharmacological blockade of dopamine receptors using agents such as haloperidol significantly suppresses environment-induced stereotyped behaviors, supporting a contributory role for dopaminergic mechanisms in their maintenance (24). Similarly, opioid receptor antagonists reduce the frequency and duration of stereotyped behaviors, suggesting that repetitive actions may generate endogenous reinforcement via intrinsic reward pathways (25).

At the molecular level, the transcription factor ΔFosB has been identified as a key mediator linking repeated neural activation to long-term neuroplastic adaptation. Sustained accumulation of ΔFosB within the striatum is a hallmark of substance-related and behavioral addictions, where it promotes compulsive behavior and diminishes behavioral flexibility (26). The widespread occurrence of abnormal repetitive behaviors across species raises the possibility that similar transcriptional mechanisms may contribute to the stabilization of stereotyped behavior (27). We propose that elevated ΔFosB expression may represent a molecular signature of long-term stereotypy, a hypothesis that warrants targeted experimental investigation.

## 5 Conclusions and implications

Recognizing stereotyped behavior as a manifestation of corticostriatal dysfunction and maladaptive neuroplasticity carries important implications for intervention strategies. Beyond conventional environmental enrichment, targeted approaches aimed at restoring behavioral flexibility and circuit balance may be required. Firstly, early-life environmental complexity during critical developmental windows may preserve corticostriatal plasticity and prevent the consolidation of maladaptive habit circuits. Secondly, combined environmental–pharmacological strategies targeting dopaminergic or glutamatergic modulation may enhance the reversibility of established stereotypy by facilitating circuit rebalancing. Thirdly, future welfare assessment frameworks may benefit from incorporating neurobiological biomarkers-such as measures of behavioral flexibility, corticostriatal connectivity, or molecular indicators of plasticity—to complement purely behavioral observations. Such integrative strategies may shift the field from reactive symptom management toward proactive preservation of neural function and adaptive capacity.
